# A prospective clinical trial of the effects produced by the extrusion arch in the treatment of anterior open bite

**DOI:** 10.1186/s40510-020-00339-z

**Published:** 2020-10-20

**Authors:** Juliana de Brito Vasconcelos, Renata Rodrigues de Almeida-Pedrin, Thais Maria Freire Fernandes Poleti, Paula Oltramari, Ana Cláudia Ferreira de Castro Conti, Mirchel Henrique Bertola Bicheline, Steven J. Lindauer, Marcio Rodrigues de Almeida

**Affiliations:** 1 Department of Orthodontics, Unniversity of North Paraná: (UNOPAR), Londrina, PR Brazil; 2 Department of Orthodontics, School of Dentistry, Virginia Commonwealth University, Richmond, VA USA

**Keywords:** Open bite, Digital models, Malocclusion

## Abstract

**Aim:**

To evaluate the maxillary dentition effects of the extrusion arch for anterior open bite (AOB) correction in mixed dentition patients.

**Materials and methods:**

Fourteen subjects with an initial mean age of 9.17 ± 1.03 years presenting with dentoalveolar AOB (mean − 1.28 ± 1.46 mm) and normal facial pattern (FMA = 25.76°) were treated with an extrusion arch. The mean treatment period was 7.79 ± 2.58 months. Lateral cephalograms and dental models were taken before (T0) and after the correction of AOB (T1). Data were analyzed using paired *t* test to evaluate differences between T0 and T1. For all tests, a significance level of *P* < .05 was used.

**Results:**

All patients achieved positive overbite at T1, with a mean increase of 3.07 mm. The maxillary incisors extruded 1.94 mm. Retroclination of the maxillary incisors (− 6.15°) and an increase in the interincisal angle (5.57°) were observed. There was a significant decrease in the distance between the incisal edge of the maxillary incisors and the molars (− 2.21 mm). There was significant mesial tipping of the maxillary molar (− 11.49°). Significant reductions of overjet (− 1.65 mm), arch perimeter (− 3.02 mm), and arch length (− 2.23 mm) were noted. The transverse maxillary intermolar distance did not change significantly.

**Conclusions:**

The use of a maxillary extrusion arch was effective in the treatment of AOB. Overbite increased due to incisor extrusion, as well as retroinclination and overjet reduction. However, side effects, such as mesial molar tipping and decreases in arch perimeter and length might occur.

## Background

Anterior open bite (AOB), defined as the absence of positive vertical overlap between the upper and lower incisors [1], is a major concern for orthodontists due to the psychological, esthetic, speech, and functional impairments it causes, in addition to having various etiologic factors [1]. Besides causing esthetic problems, function, phonation and breathing may become impaired due to consequent changes in the balance of the stomatognathic system.

Several factors may be involved in developing and maintaining AOB, including skeletal, dental, and functional factors and especially the presence of deleterious oral habits [2, 3]. Therefore, the cause of persistent AOB is likely multifactorial due to the interaction of several factors [1]. Among the oral habits most often associated with this malocclusion are thumb and pacifier sucking [3, 4, 5]. Due to this strong association with the occurrence of oral habits, it is more prevalent during childhood, reaching 17% of the population during the mixed dentition phase [6, 7].

Different treatment modalities have been recommended with appliances such as fixed and removable palatal cribs, bonded spurs, chincup [5, 8, 9], and intermaxillary elastics being commonly used [10]. Despite having excellent results, the majority of these appliances require patient cooperation and can cause some discomfort. Thus, approaches with fixed devices that do not rely on patient compliance have been increasingly adopted, such as the extrusion arch [10, 11, 12].

The extrusion arch is an effective option for maxillary incisor extrusion and treatment of dentoalveolar AOB [10, 11, 12, 13]. The activation of the arch is the opposite of the intrusion arch, with an asymmetrical V-shape bend exerting a force around 40–60 g [10, 13] on the anterior teeth. It is a predictable one-couple system that generates extrusive force on the incisors, promoting bite closure [10, 12], improving function and esthetics.

There have been no prospective clinical studies that investigated the effects of AOB treatment using a maxillary extrusion arch published in the literature. Thus, the aim of the present study was to evaluate the effects caused by the use of an extrusion arch in the early treatment of AOB by means of lateral cephalograms and dental models. Based on the concepts of static equilibrium, the hypothesis was that the extrusion arch would achieve successful closure of AOB by incisor extrusion with predictable side effects of molar anterior tipping and incisor uprighting.

## Methods

This was a prospective study approved by the review board of the institution. Patients and guardians were fully informed about the study and its implications and written consent was obtained.

STROBE (STrengthening the Reporting of OBservational studies in Epidemiology) statement guidelines for observational studies were followed. The clinical trial was conducted from March 2018 to December 2019. Sample size estimation was performed based on a significance level of 5% (alpha) and a beta value of 0.2, to achieve a minimum of 80% probability of detecting a mean difference of 1.5 mm in overbite with an estimated standard deviation of 1.69 [14]. A minimum of 12 patients were required.

**Fig. 1:**
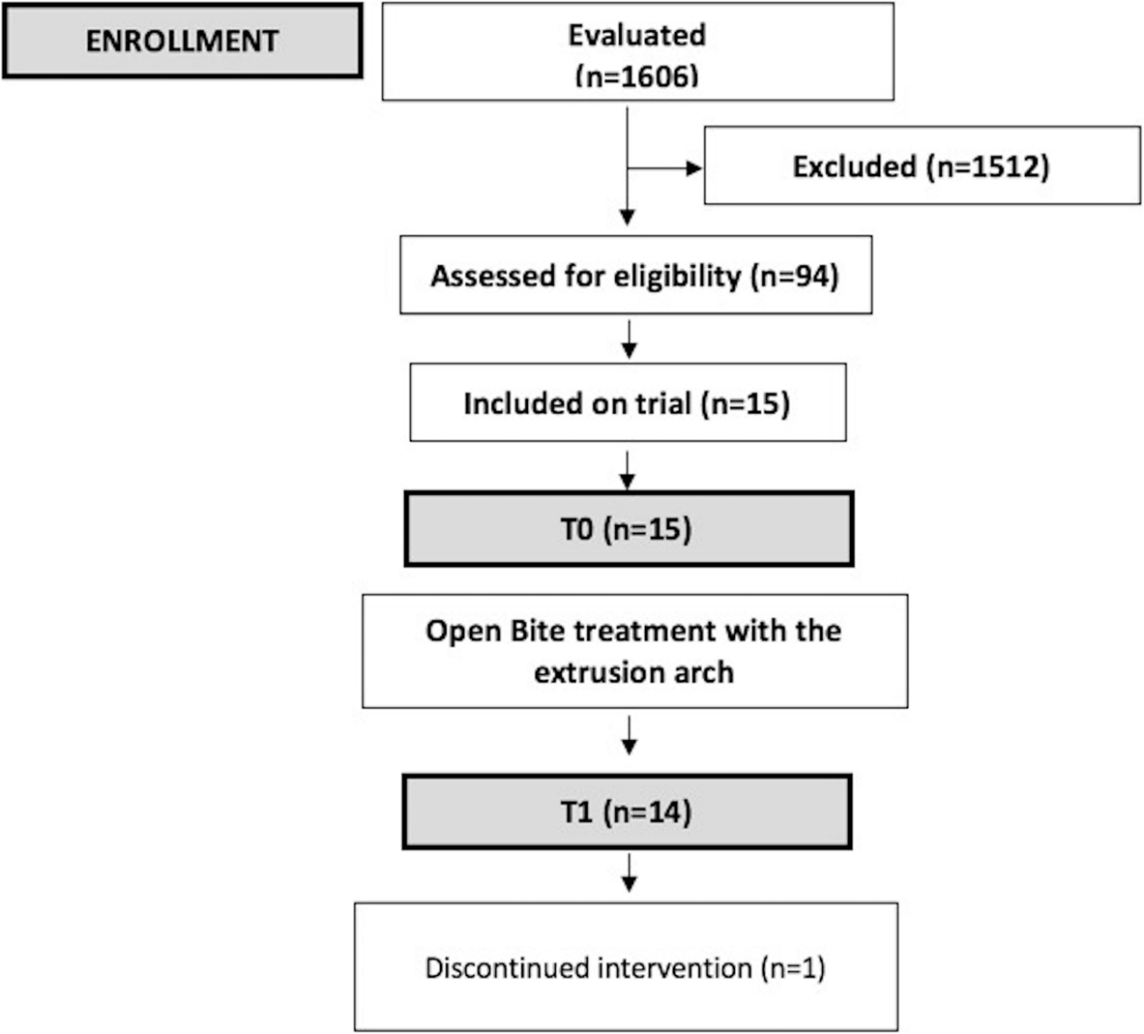
Consolidated Standards of Reporting Trials (CONSORT) flow diagram

As shown by the flow chart (Fig. 1), 1606 children were initially evaluated in municipal schools. After initial evaluation, 94 children were selected according to the following inclusion criteria: individuals in the mixed dentition phase who displayed a dentoalveolar anterior open bite (AOB equal to or greater than 0 mm), normal facial pattern**,** first permanent molars in occlusion, class I molar relationship, without permanent tooth loss and good oral health. Those who presented with a skeletal open bite (hyperdivergent phenotype), posterior crossbite, syndromes, trauma to the maxillary incisors, skeletal asymmetries, patients in need of extractions, patients with significant crowding in the maxillary arch (> 3 mm), agenesis (except for third molars), or dental anomalies were excluded. Fifteen patients agreed to participate in the research and over the course of the study, one patient was lost. The remaining 14 patients completed the study.

### Extrusion mechanics and guidelines

The same treatment protocol was followed for all patients. Conventional brackets (Roth prescription, 0.022 × 0.028-in. slot, Orthometric, Marília, SP, Brazil) were bonded to the maxillary incisors. Orthodontic bands were cemented to the maxillary first molars with double tubes. A passive transpalatal bar for anchorage was adapted to the first molars.

**Fig. 2:**
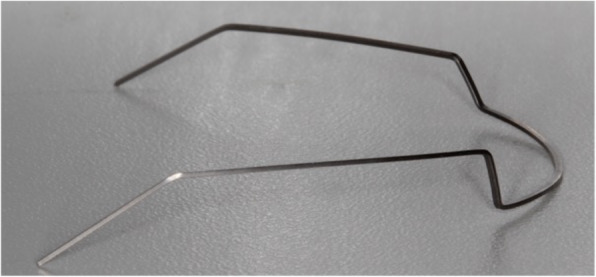
Extrusion arch constructed of 0.017“x0.025” TMA wire

**Fig. 3:**
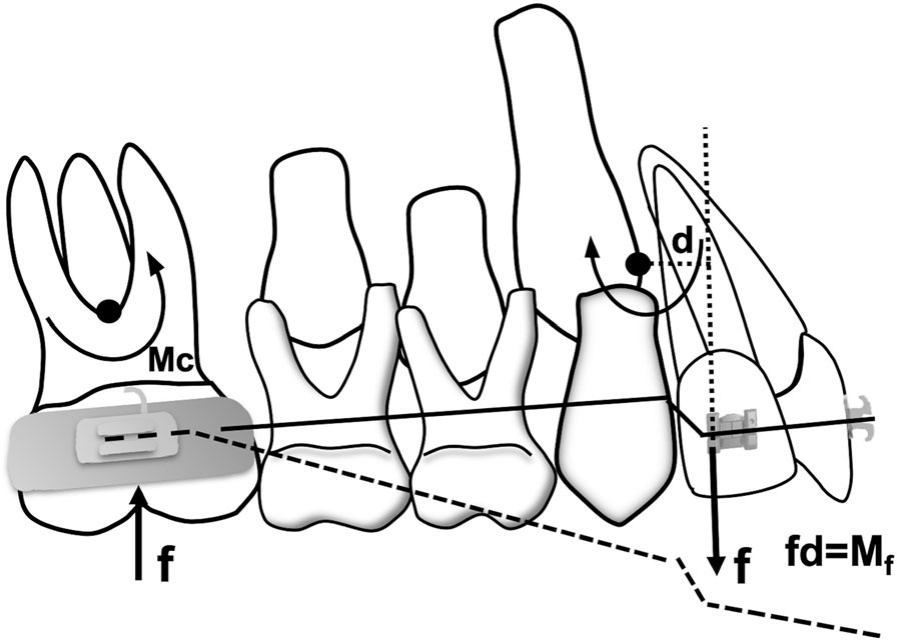
Schematic drawing of the Extrusion arch force system

An extrusion arch made of beta-titanium alloy 0.017 × 0.025-inch (Orthometric, Marília, SP, Brazil) (Fig. 2) was hand bent and placed in the maxillary first molar auxiliary tubes and tied over a segment of 0.014 × 0.025-in. Copper-NiTi on the maxillary lateral incisors brackets secured by a metal ligature. From the biomechanical standpoint, it should be mentioned that all patients received extrusion arches that were attached to the distal surface of the maxillary lateral incisors brackets in order to provide appropriate point of force application as well as correct moment to force ratio while applying the concept of a one couple force system (Fig. 3).

**Fig. 4:**
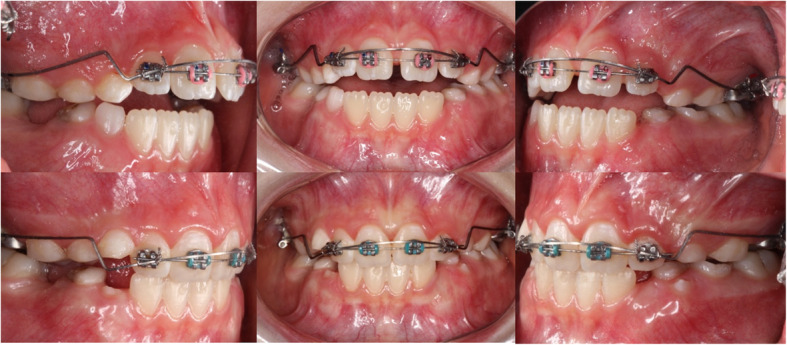
Patient with an AOB showing the mechanics of the extrusion arch before and after 6 months of treatment

The extrusion arch was cinched-back on the distal surface of the tube of molars. The arch was activated with a V-bend located 1–3 mm mesial to the maxillary molars and calibrated every month with a tension gauge to deliver an extrusive vertical force of approximately 40–60 g. The extrusion arch remained until the correction of the AOB (Fig. 4). Reactivation of the extrusion arch was not necessary, since the range of the force was kept within 40–60 g during the entire period of the study. All patients were treated by only one operator and appointments were made every month. No appliance breakages were reported for the 14 patients.

### Lateral cephalometric and digital model analysis

**Fig. 5:**
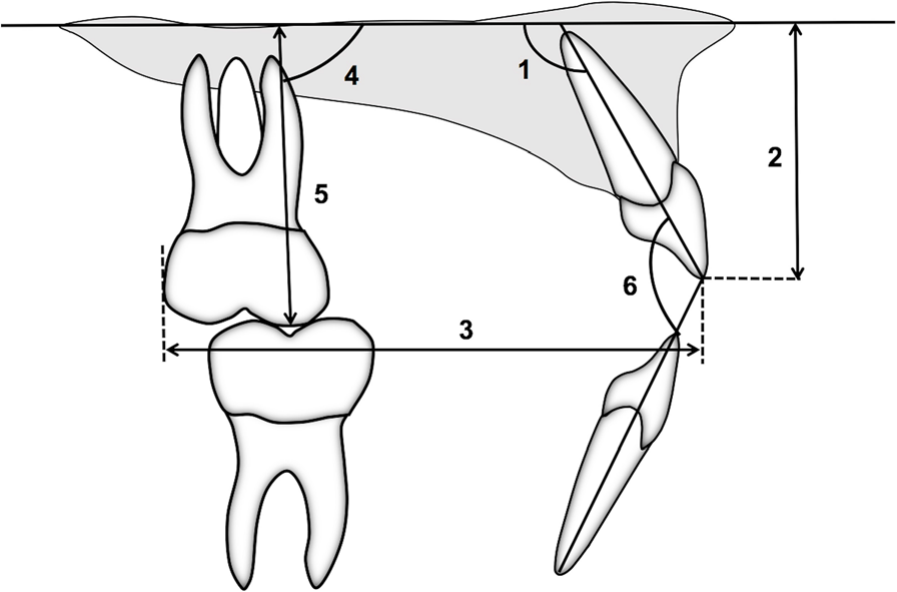
Cephalometric diagram used for cephalogram measurements: PP: palatal plane, horizontal reference line passing from anterior nasal spine (ANS) to posterior nasal spine (PNS); 1, U1.PP: angulation of maxillary central incisor to PP; 2, U1-PP: linear distance of maxillary incisor incisal edge to PP; 3, U1tip to U6D: linear distance of maxillary incisor tip to maxillary first molar distal aspect perpendicular to PP; 4, U6.PP^o^: angular measurement determined by a line passing through the mesio-buccal cusp and root apex, perpendicular to maxillary first molar mesial surface to PP; 5, U6-PP: distance of maxillary first molar mesio-buccal cusp to PP; 6, 1.1: Interincisal angle: angle formed between maxillary and mandibular incisor long axes

**Fig. 6:**
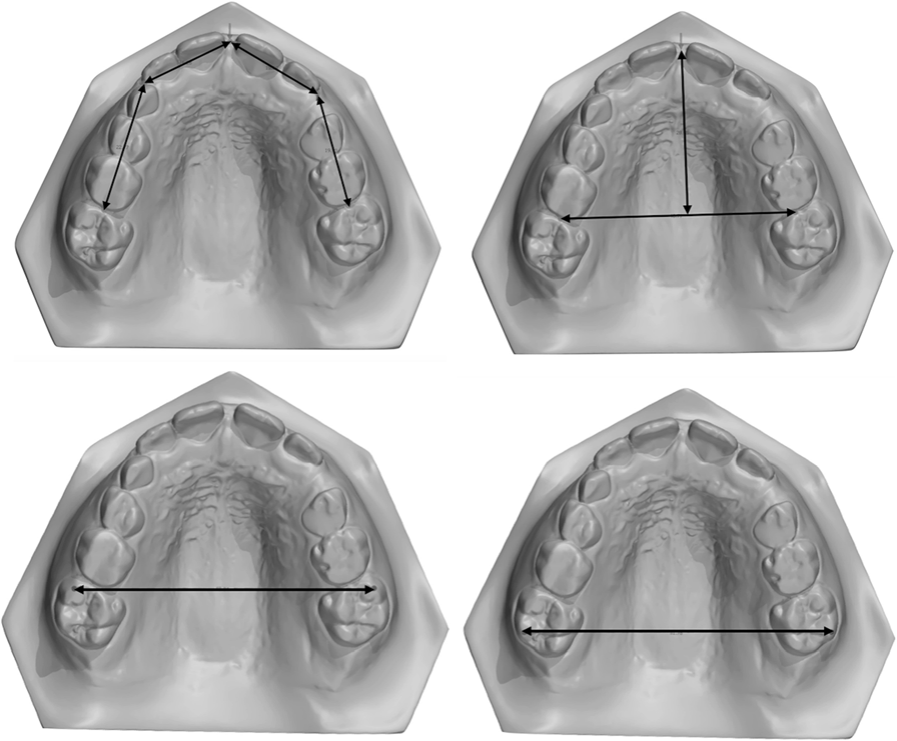
Evaluation of arch perimeter, arch length and intermolar distance at mesial and distal cusps

**Fig. 7:**
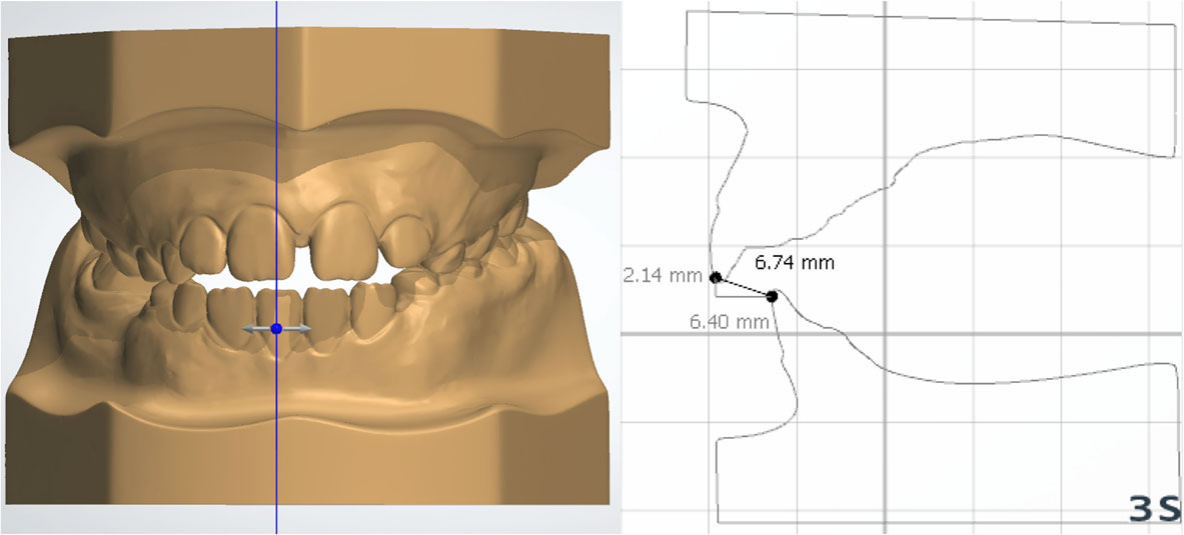
Measurement of overbite and overjet

**Fig. 8:**
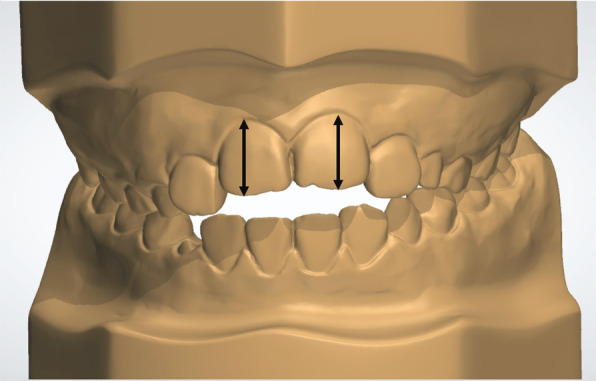
Maxillary Incisor clinical crown heights

The patients were analyzed at two time points: initial (T0) and after correction of the AOB (T1). Lateral cephalometric radiographs from all patients were traced by a single calibrated examiner with the aid of Dolphin^TM^ Imaging software (version 11.7, Dolphin Imaging and Management Solutions, Chatsworth, CA, USA). The palatal plane was used as a reference (horizontal reference line passing from anterior nasal spine [ANS] to posterior nasal spine [PNS]) and six linear and angular measures regarding the molars and incisors were made (Fig. 5). Conventional plaster models were obtained at T0 and T1 and digitized by a 3Shape R700 3D scanner (3Shape A/S, Copenhagen, Denmark) to facilitate analysis with OrthoAnalyzer software (3Shape A/S, Copenhagen, Denmark). Maxillary dimensional changes were evaluated regarding the following parameters [15]: arch perimeter and length, overjet, overbite, intermolar distances, and maxillary incisor clinical crown height (Figs. 6, 7, and 8).

### Statistical analysis and error of the method

Data distribution was analyzed by the Kolmogorov-Smirnov normality test. A normal distribution of data was found. For comparison between the initial (T0) and final (T1) time points, paired *t* tests were used. Reliability was assessed by repeating cephalometric and model measurements for 30% of the sample after 30 days. The results were analyzed by intraclass correlation coefficient (ICC), Dahlberg test, paired *t* test, Bland-Altman, and Person correlation coefficient. All statistical procedures were performed with the aid of Statistica 5.0 software (StatSoft Inc., Tulsa, USA). Significance level was set at 5%.

## Results

The characterization of the sample in relation to gender distribution, treatment time and mean age are shown in Table 1. The sample had a mean FMA of 25.76°. Regarding the frequency of habits among patients, 70% reported a history of pacifier sucking used in the past.

**Table 1:** Description of sample characteristics

Gender	Female	8 (57%)
	Male	6 (43%)
Treatment time	Mean	SD
(months)	7.79	2.58
Mean age (years)	Mean	SD
	9.17	1.03

Intra-examiner reliability of the cephalometric measurements was excellent (Dahlberg test ranged from 0.19 mm to 0.75°) (ICC ranged from 0.92 to 1.00). None of the cephalometric variables showed a significant systematic error as assessed by paired *t* test. Regarding the digital model analysis, high reliability was found (ICC ranged from 0.95 to 1). The Bland-Altman test showed a low degree of bias for most of the repeated measures, with the Pearson correlation coefficient ranging from 0.97 to 1 and the confidence intervals ranging from − 0.020 to 0.937 for the upper limit and from − 1.317 to 0.018 for the lower limit.

Table 2 shows the data obtained by digital model evaluation. Significant reductions in arch perimeter (− 3.02 ± 3.07 mm), arch length (− 2.23 ± 1.85 mm) and overjet (− 1.65 ± 1.75 mm) were observed. Pre- and post-treatment overbite were − 1.28 ± 1.46 mm and 1.79 ± 1.23 mm, respectively. A significant increase of 3.07 ± 1.57 mm was found in the overbite. The maxillary intermolar transverse measures did not show significant changes. When evaluating the heights of the clinical crown of the incisors, a statistically significant increase of 0.33 ± 0.64 mm was observed.

**Table 2:** Mean values with standard deviations and 95% confidence intervals (CI) of dimensional dental casts variables at T0 and T1 achieved with the extrusion arch mechanics with mean differences^a^

Variables	T0 (Initial)		T1 (Final)		T1–T0		CI (95%)		
	Mean	SD	Mean	SD	Mean	SD	UB	LB	*P* value
Arch Perimeter	78.66	4.84	75.63	5.86	− 3.02	3.07	− 4.79	− 1.25	.003*
Arch Length	29.07	2.46	26.84	2.92	− 2.23	1.85	− 4.79	− 1.16	.001*
Overjet	4.89	2.01	3.24	1.47	− 1.65	1.75	− 2.65	− 0.64	.004*
Overbite	− 1.28	1.46	1.79	1.23	3.07	1.57	− 2.17	− 3.97	< .0001*
Mesial intermolar distance	51.65	3.08	51.31	2.85	− 0.33	0.90	− 0.85	0.18	.188
Distal intermolar distance	55.05	3.49	54.69	3.66	− 0.36	1.08	− 0.98	0.27	.239
Incisors clinical crown height	9.11	0.74	9.44	0.92	0.33	0.64	− 0.08	0.58	.011*

^a^*LB* indicates lower bound, *UB* upper bound, *SD* indicates standard deviation

*P < .05

Table 3 presents the results of the cephalometric evaluation of the patients between T0 and T1. An increase in the interincisal angle (5.57^°^ ± 6.59^°^) and retroclination of the maxillary incisors (− 6.15^°^ ± 6.42^°^) were observed. Significant maxillary incisor extrusion, measured as the vertical change in position of the incisal edge (1.94 ± 1.71 mm) was noted. There was a significant decrease in the distance between the incisal edge of the maxillary incisors and the molars (^°^2.21 ± 2.34 mm). No significant difference was found regarding the vertical position of the maxillary molar. However, there was significant mesial tipping of maxillary molars (^°^11.49^°^ ± 8.41^°^).

**Table 3:** Mean values with standard deviations and 95% confidence intervals (CI) of variables at T0 and T1 achieved with the extrusion arch mechanics with mean differences (T1–T0)^a^

Variable	T0 (initial)		T1 (Final)		T1–T0		CI (95%)		
	Mean	SD	Mean	SD	Mean	SD	UB	LB	*P* value
Interincisal angle (°)	115.58	7.04	121.15	10.57	5.57	6.59	1.77	9.38	.010*
U1.PP (°)	115.73	4.40	109.58	5.96	− 6.15	6.42	− 9.85	− 2.44	.003*
U1-PP (mm)	25.45	3.38	27.39	2.77	1.94	1.71	0.96	2.93	.001*
U1 tip-U6D (mm)	40.82	2.56	38.61	3.65	− 2.21	2.34	− 3.57	− 0.84	.004*
U6-PP (mm)	17.86	1.94	16.85	2.38	− 1.01	3.22	− 2.86	0.85	.260
U6.PP (°)	86.56	10.47	75.07	7.26	− 11.49	8.41	6.63	16.34	< .0001*

^a^*LB* indicates lower bound, *UB* upper bound, *SD* indicates standard deviation, *U1* maxillary central incisor, *U6* maxillary first molar, *U6D* distal aspect of maxillary first molar

*P < .05

## Discussion

The extrusion arch has been suggested as a predictable one-couple appliance that can be used for the treatment of AOB without the need for patient compliance [10, 12]. No prospective clinical study on its use has been previously published and therefore, there was no attempt to include to the present study a different treated group of patients, i.e., spurs and/or palatal cribs. Our main objective of this prospective clinical study was to quantify the relative maxillary incisor and molar movements in AOB patients treated using these mechanics by means of lateral cephalograms and cast models. The hypothesis of the study was accepted since the extrusion arch achieved successful closure of AOB by maxillary incisor extrusion with predictable side effects of molar anterior tipping and incisor uprighting. Our hypothesis is that with an extrusion arch the correction of AOB occurs within 5 to 8 months due to the forced extrusion of maxillary incisors. No spurs or tongue crib were used in our patients in order to interrupt sucking and thrusting habits, which may allow normal vertical development at the anterior region by elimination of the tongue contact.

Extrusion arch mechanics produced a mean maxillary incisor extrusion of 1.94 mm during a mean treatment period of 7.79 months. Previous studies observed similar results with other types of appliances over a 12-month period [8, 9, 14, 16, 17]. The removable palatal crib (RPC) resulted in incisor extrusion ranging from 1.64 mm [8], 2.47 mm [9], and 2.98 mm [17], while the use of bonded spurs (BS) promoted changes of 1.50 mm [8], 2.35 mm [9], 3.16 mm [16], and 2.33 mm [17] when associated with a chincup (CC) [16].

In the current study, overbite increased 3.07 mm in 7.79 months, with a final mean overbite of 1.79 mm. A previous study observed 3.51 mm of overbite correction and a final overbite of 0.57 mm with the use of fixed palatal crib (FPC) and 3.88 mm of correction and 0.84 mm of final overbite with the FPC over a period of 1 year [15]. These minor differences can be explained by the shorter treatment time of the present study and the fact that the use of the palatal crib also helped with the postural reeducation of the tongue, which does not occur with the extrusion arch mechanics. On the basis of these findings, it could be recommended that the extrusion arch be supplemented with appliances designed to alter tongue positioning in patients with a tongue thrust habit after the closure of AOB is accomplished.

In addition to the extrusive vector, the one-couple mechanics produced an uprighting moment on the maxillary incisors [10, 12]. This effect is favorable in most cases of AOB, which are usually accompanied by incisor labial proclination. In the present study, there was significant palatal tipping of the maxillary incisors (− 6.15 ± 6.42°). A previous study [18] observed a non-significant palatal tipping of the incisors with the use of palatal cribs (− 1.77°) and spurs (− 4.1°). Also, in the current study, a significant increase of 5.57° in the interincisal angle was observed, due to the retroclination of the anterior teeth. Similarly, previous studies observed an increase in the interincisal angle ranging from 9.66° (palatal crib and chincup) [16], 3.34° (BS), 9.65° (FPC), 7.01° (RPC), and 4.25° (CC) [8].

As a consequence of incisor retroclination, a significant reduction (− 1.65 mm) of overjet was also observed. These results were in contrast to those of previous studies [15, 16] which showed no significant differences in the overjet in patients treated with the RPC and FPC [15, 16]. In addition to overjet reduction, arch perimeter and length were also significantly reduced (− 3.02 mm and − 2.23 mm, respectively). These results were similar to a previous study [15], where an arch perimeter reduction of 0.92 mm and 0.38 mm and 1.34 mm and 0.52 mm arch length reduction were reported, with the use of the RPC and FPC, respectively. Additionally, it was shown that the palatal crib produced arch perimeter and length reductions of 2.6 mm and 1.4 mm, respectively [19]. In the present study, the arch perimeter and length reductions were greater in magnitude compared to previous studies. The most likely explanation for this finding was that the extrusion arch exerted a force on the maxillary incisors located anterior to the center of resistance, thus resulting in a moment tending to upright the anterior teeth. The palatal crib or spurs, on the other hand, promoted only passive extrusion of the incisors due to the habit cessation and postural reeducation of the tongue and upper lip posture, thus allowing extrusive movement of the incisors without a significant decrease in arch length or perimeter.

During extrusive movement of the incisors, the periodontium (gingiva) may not follow the teeth, with a possible increase in the clinical crown height. A small, but statistically significant increase of 0.33 mm in the incisor clinical crown height was observed. However, this was not considered to be clinically significant because this minor increase does not compromise periodontal integrity. Also, this change may be expected since mixed dentition patients often exhibit an under development of the vertical position of the maxillary incisors prior to AOB closure. Unfortunately, no other previous study investigated the amount of vertical changes in relation to the gingiva and clinical crown height occurred during the use of an extrusion arch.

In contrast to the extrusive force vector on the incisors, an intrusive force was generated on the molars. This would be expected to occur as a side effect of the one-couple force system [12]. A non-significant intrusion of − 1.01 mm was observed at the molars. This was contrary to previous studies which observed molar extrusion while using CC [8, 14, 17], BS, and PC [12]. A more unfavorable movement of the maxillary molars was due to the counter-clockwise couple produced as part of the extrusion arch force system, which promoted anterior tipping of the molars [10, 12]. Significant maxillary molar mesial inclination of 11.49° was observed. This side effect also contributed toward the reductions in arch length and perimeter, even though a transpalatal bar was used in all patients. The reduction in arch length and perimeter might cause some loss of space for maxillary permanent canines’ eruption, which called for attention during the transition to the permanent dentition. Based on these findings, care should be taken in order to avoid these effects if mesial inclination or intrusion of the maxillary molars is not desired. Perhaps, bonding the posterior deciduous teeth and using a rigid buccal segment archwire to reinforce anchorage might prevent these side effects during use of the extrusion arch. In order to eliminate the mesial maxillary molar inclination, temporary anchorage device such as miniscrews to serve as indirect anchorage could also be used. Another way to reduce side effects on the maxillary molars would be to decrease the extrusive force of the extrusion arch. As mentioned before, our extrusion arch made of beta-titanium alloy 0.017 × 0.025-in. delivered 40–60 g of force on the anterior teeth, which can be considered by some authors too high. Uribe et al. [20] advocated lower forces (30–40 g) in order to minimize side effects on maxillary molars. Thus, Uribe et al. [20] indicated to insert the Connecticut intrusion arch upside-down, which is a preformed archwire made of nickel-titanium exerting 35 g of force in order to treat an AOB.

No significant changes in the transverse dimension were observed in the present study. This result was in contrast to that observed for palatal cribs, which showed a significant increase in the transverse dimension [15]. In the present study, a transpalatal bar might have maintained the intermolar distance during AOB closure.

Overall, the extrusion arch showed predictable results, closing the AOB in 100% of the patients. Overbite correction occurred mainly from maxillary incisor extrusion and retroclination. Mesial tipping of the maxillary molars due to the counter-clockwise couple produced by the appliance should be monitored.

### Limitations

Some limitations of this study included the severity of the anterior open bite in the patients enrolled and the short-term evaluation period. It is important to recognize that the results reported were obtained when treating a sample of dental AOB malocclusions; thus, they cannot be extrapolated to skeletal AOB subjects. Long-term studies are also recommended to assess potentially increasing posterior crowding and stability of the results.

## Conclusions

1. The extrusion arch produced an AOB closure in all patients with a mean overbite correction of 3.07 mm in a mean period of 7.79 months.
2. Retroclination of the maxillary incisors, a reduction in the overjet, and decreases in the perimeter and arch length occurred during the use of the extrusion arch.
3. A counter-clockwise couple with resultant mesial tipping of the maxillary molars should be expected as a side effect of this one-couple system appliance.
4. The tooth movements observed were consistent with those that would be predicted by biomechanical analysis of the one-couple force system exerted by the extrusion arch appliance.

## Data Availability

Please contact the corresponding author for data requests.

## Abbreviations

AOB: Anterior open bite
STROBE: STrengthening the Reporting of OBservational studies in Epidemiology
ANS: Anterior nasal spine
PNS: Posterior nasal spine
ICC: Intraclass correlation coefficient
RPC: Removable palatal crib
BS: Bonded spurs
CC: Chincup
FPC: Fixed palatal crib
